# Assessing ChatGPT’s Competency in Addressing Interdisciplinary Inquiries on Chatbot Uses in Sports Rehabilitation: Simulation Study

**DOI:** 10.2196/51157

**Published:** 2024-08-07

**Authors:** Joseph C McBee, Daniel Y Han, Li Liu, Leah Ma, Donald A Adjeroh, Dong Xu, Gangqing Hu

**Affiliations:** 1 Department of Microbiology, Immunology, & Cell Biology West Virginia University Morgantown, WV United States; 2 Department of Chemical and Biomedical Engineering West Virginia University Morgantown, WV United States; 3 College of Health Solutions Arizona State University Phoenix, AZ United States; 4 Biodesign Institute Arizona State University Tempe, AZ United States; 5 College of Health, Education, and Human Services Wright State University Dayton, OH United States; 6 Lane Department of Computer Science & Electrical Engineering West Virginia University Morgantown, WV United States; 7 Department of Electrical Engineering and Computer Science Christopher S. Bond Life Sciences Center University of Missouri Columbia, MO United States

**Keywords:** ChatGPT, chatbots, multirole-playing, interdisciplinary inquiry, medical education, sports medicine

## Abstract

**Background:**

ChatGPT showcases exceptional conversational capabilities and extensive cross-disciplinary knowledge. In addition, it can perform multiple roles in a single chat session. This unique multirole-playing feature positions ChatGPT as a promising tool for exploring interdisciplinary subjects.

**Objective:**

The aim of this study was to evaluate ChatGPT’s competency in addressing interdisciplinary inquiries based on a case study exploring the opportunities and challenges of chatbot uses in sports rehabilitation.

**Methods:**

We developed a model termed PanelGPT to assess ChatGPT’s competency in addressing interdisciplinary topics through simulated panel discussions. Taking chatbot uses in sports rehabilitation as an example of an interdisciplinary topic, we prompted ChatGPT through PanelGPT to role-play a physiotherapist, psychologist, nutritionist, artificial intelligence expert, and athlete in a simulated panel discussion. During the simulation, we posed questions to the panel while ChatGPT acted as both the panelists for responses and the moderator for steering the discussion. We performed the simulation using ChatGPT-4 and evaluated the responses by referring to the literature and our human expertise.

**Results:**

By tackling questions related to chatbot uses in sports rehabilitation with respect to patient education, physiotherapy, physiology, nutrition, and ethical considerations, responses from the ChatGPT-simulated panel discussion reasonably pointed to various benefits such as 24/7 support, personalized advice, automated tracking, and reminders. ChatGPT also correctly emphasized the importance of patient education, and identified challenges such as limited interaction modes, inaccuracies in emotion-related advice, assurance of data privacy and security, transparency in data handling, and fairness in model training. It also stressed that chatbots are to assist as a copilot, not to replace human health care professionals in the rehabilitation process.

**Conclusions:**

ChatGPT exhibits strong competency in addressing interdisciplinary inquiry by simulating multiple experts from complementary backgrounds, with significant implications in assisting medical education.

## Introduction

The sports industry is a significant economic contributor in the United States, which was projected to generate US $83.1 billion in revenue in 2023 [1]. Concurrently, sports/recreation-related injuries are prevalent, with an estimated rate of 34 per 1000 individuals, accumulating to an annual total of 8.6 million cases [2]. Sports rehabilitation, aiming to facilitate full recovery, minimize sports downtime, and prevent future injuries, is a process of coordinated efforts between the athlete and health care professionals across various disciplines [3]. However, the rehabilitation process often spans a lengthy period and demands expensive medical and psychological support, making it inaccessible for many patients. In recent years, the integration of artificial intelligence (AI) in sports medicine has shown promise in enhancing both the accessibility to service and the efficacy of treatment outcomes [4,5]. Nevertheless, the use of chatbots in assisting sports rehabilitation is still in its formative stages, with many potential benefits and pitfalls yet to be explored and understood.

ChatGPT, a sophisticated large language model (LLM)–based chatbot, is capable of human-like dialogue [6]. This chatbot exhibits promise as a virtual assistant in medical education by providing real-time personalized feedback and enhancing student engagement [7]. However, controlled assessments in medical education have identified considerable limitations such as the need for precise prompts (also known as prompt engineering), instances of hallucination, and a lack of critical thinking in its responses [8-10]. Another challenge is that many of the topics in health care are interdisciplinary, involving multiple contributors such as physicians, pharmacists, and social workers to ensure better treatment outcomes and patient satisfaction. Unfortunately, current evaluations of ChatGPT are often confined to tasks from a specifical discipline, leaving its competency in addressing interdisciplinary topics largely unexplored [11,12], especially in medical education fields such as sports rehabilitation [5,13].

Here, we highlight an attractive feature of ChatGPT in addressing interdisciplinary questions via multirole-playing, which allows the chatbot to assume the roles of several discipline-specific experts simultaneously in one chat session. This unique feature inspired us to propose a model named PanelGPT for exploring interdisciplinary topics through a simulated panel discussion, where ChatGPT assumes the roles of a moderator and various experts on the panel. The aim of the study was to evaluate ChatGPT’s competency through PanelGPT in addressing the opportunities and challenges of chatbot uses in sports rehabilitation, an interdisciplinary field that covers topics on patient education, physical therapy, psychological support, nutrition, and ethics.

## Methods

### The PanelGPT Model

**Figure 1:**
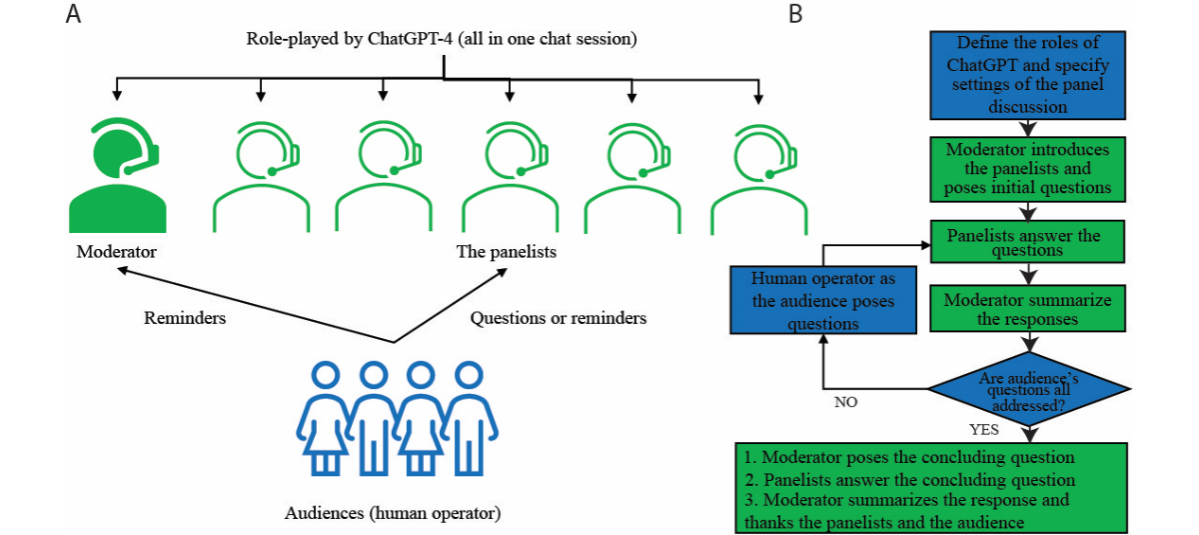
Overview of the PanelGPT model for a ChatGPT-simulated panel discussion (A) and a flowchart that delineates the simulation process (B).

We developed a model named PanelGPT to evaluate ChatGPT’s competency in addressing interdisciplinary inquiry (Figure 1A). In this model, ChatGPT assumes the roles of both the moderator and panel experts, while a human operator, representing the audience, poses questions and sends reminders to the moderator or the panelists. Questions from the human operator are directly copied and pasted into the chat session, with ChatGPT determining which panel member(s) should respond. If the discussion stalls, the human operator prompts the moderator or panelists to continue by sending reminders. After each round of discussion, the moderator summarizes the comments before moving to the next question from the audience. Upon conclusion of the discussion, we summarized and evaluated the chatbot's responses based on the literature and our expertise.

### Application to Chatbots in Sports Rehabilitation

We applied PanelGPT to explore the pros and cons of chatbot uses in sports rehabilitation. The simulated panelists included 4 experts representing essential disciplines related to the topic: a physiotherapist, psychologist, nutritionist, and AI expert specializing in clinical applications. In addition, a virtual athlete who had successfully recovered from a severe injury participated in the panel. We formulated 4 main questions based on personal experience and/or a literature review. After reviewing the responses from pilot simulations, we added 2 more questions (Multimedia Appendix 1 [14-16]). During one of the pilot simulations, ChatGPT autonomously introduced opening questions, which we subsequently included in the final simulations. This finding also inspired us to instruct the chatbot to ask closing questions at the end of each simulation.

To clarify, our focus is not on using ChatGPT to provide sports rehabilitation advice. Instead, we centered on using ChatGPT to drive a panel discussion titled “Chatbots in sports rehabilitation” in a “self-consistency” manner [17]. The prompts used to steer the final simulations are detailed in Multimedia Appendix 2. A flowchart that outlines the process of the simulation is shown in Figure 1B. At the beginning of the prompts, we instructed ChatGPT to undertake multiple roles and specified other settings in the simulation (Multimedia Appendix 2). Next, the moderator was prompted to introduce the panelists and kick off the discussion with opening questions. Following the responses to these initial questions from the panelists, the moderator was tasked to summarize the responses and open the platform for questions from the audience. In response, the human operator copied each question from the audience directly into the chat session, allowing ChatGPT to select which expert should respond autonomously. After each round of questions and answers, the moderator was prompted to summarize the responses and call for the next question. This process was iterated until all of the audience’s questions had been addressed. At the end of the panel discussion, the moderator was asked to propose a closing question and provide a summary of the responses. Additional prompts were introduced as needed to ensure a smooth progression of the panel discussion (Multimedia Appendix 2). We repeated the simulation 3 times using ChatGPT-4 (May 24, 2023, version) with its online web interface [18].

As shown in Multimedia Appendix 1, we initiated the simulation with an opening question and concluded with a closing question. During the simulation, we prompted ChatGPT to simulate a panel discussion on topics from chatbot uses in sports rehabilitation in the order of “patient education,” “physical therapy,” “psychological support,” “nutrition,” “tracking & other alternatives,” and “ethics.” After 3 rounds of simulations, we manually evaluated the panel’s response to questions from each topic by referring to the literature and our human expertise.

### Ethical Considerations

This work was based on analyzing ChatGPT’s response to designed prompts. As the work is classified as not human subjects research, review of the Institutional Review Board of West Virginia University was not required [19].

## Results

### Overview

The complete chat histories, including prompts and ChatGPT’s response from the simulated panel discussion, are accessible in Multimedia Appendices 3-5 (audio versions are available upon request). As expected, 2 or more experts responded to each question (Table 1); the experts generally offered insights from their respective fields of expertise. We evaluated the responses by citing relevant references and according to our own expertise. The most relevant findings are compiled and summarized below for each question.

**Table 1:** Records of direct responses to questions during the simulation.^a^

Question	Physiotherapist	Psychologist	Nutritionist	Athlete	AI^b^ expert
Opening question	1, 2, 3	1, 2, 3	1, 2, 3	1, 2, 3	1, 2, 3
Patient education	1, 2, 3	1, 2, -	-, -, -	1, -, 3	1, 2, 3
Physical therapy	1, 2, 3	-, -, -	-, -, -	-, -, -	1, 2, 3
Psychological support	-, -, -	1, 2, 3	-, -, -	1, -, -	1, 2, 3
Nutrition	-, -, -	-, -, -	1, 2, 3	-, -, -	1, 2, 3
Tracking and other alternatives	1, 2, 3	1, 2, -	-, -, -	1, 2, -	1, 2, 3
Ethics	1, 2, 3	1, 2, -	1, 2, -	1, -, -	1, 2, 3
Closing question	1, 2, 3	1, 2, 3	1, 2, 3	1, 2, 3	1, 2, 3

^a^Numbers 1, 2, and 3 indicate when a response directly targeting the question was made for rounds 1, 2, and 3 of the simulation, respectively, whereas “-” denotes the absence of such a response.

^b^AI: artificial intelligence.

### Opening Question

The simulated panel discussion began with introductions and requests for the panelists’ perspectives on the role of chatbots in sports rehabilitation, to which all panel members responded (Table 1). The ensuing dialogue identified chatbots as round-the-clock support systems, adept at monitoring, offering reminders, consulting, and nurturing a positive mindset in athletes during their recovery. Similar observations have been reported for orthopedic patients with AI assistance in the literature [14,20,21]. Looking into the future and consistent with expectations, chatbots might grow increasingly adept at analyzing biomechanical data, emotional indicators, and nutritional needs, thus providing personalized feedback that helps athletes better comprehend their bodies and healing journeys.

### Patient Education

The conversation pinpointed several critical factors in educating athletes on using chatbots for rehabilitation. Both the athlete and the psychologist touched on the importance of understanding the benefits of using a chatbot, such as a readily available source of advice and mental support [22]. The AI expert emphasized the education on transparency, including how data are collected, processed, stored, and protected. Effective communication with a chatbot is a nontrivial task [23]. The physiotherapist focused on how to guide users to interact with the chatbot effectively and how to interpret the responses. The discussion also underscored that the chatbot system is designed to enhance recovery, not to replace the human touch. Through education, athletes need to be able to identify situations that call for direct communication with health care professionals.

### Physical Therapy

The primary focus of these questions was on the chatbot’s potential to facilitate physical therapy by analyzing movements and weight distributions [15]. Relevant responses were from the physiotherapist and the AI expert, who acknowledged that current chatbots primarily interact with users through text and voice, which restricts their direct applicability to the question. However, the AI expert envisioned integrating chatbots, wearables, cameras, and smart devices to analyze an athlete’s movement patterns and provide real-time, personalized feedback. A good example, as has also been noted in the literature, is computer vision–based analysis that has been applied to monitor and improve sports performance [24]. The AI expert further highlighted that the accuracy of this application depends on the size and quality of the training data, as well as advances in AI technologies such as machine learning and computer vision.

### Psychological Support

This round of discussion explored the role of chatbots in analyzing emotional cues via sentiment analysis, a technique previously shown to enhance patient satisfaction in several medical chatbot applications [16,25,26] and other applications [27]. The panel’s responses aligned with the existing literature: by delivering tailored responses to emotions, chatbots offer athletes emotional support and reduce their feelings of isolation. Nevertheless, the panel did not explore the impact of chatbots on psychological outcome measures such as improvements in communication skills, cognitive level, motivation, and abilities in coping with the injury. The psychologist and the AI expert cautioned that sentiment analysis may not always capture human emotions accurately. Thus, the psychological support provided via chatbots should be regarded as a complement to human interventions, which, in our opinion, can extend from health care professionals to coaches, teammates, friends, and family members.

### Nutrition

Chatbots have been used for nutrition advice [28-30]. The nutritionist outlined multiple roles for chatbots in nutritional management, such as reminding athletes to stay hydrated, tracking dietary intake, and suggesting meal plans. A personalized dietary plan could use an advanced AI algorithm to analyze factors such as demographics, injury type, recovery stage, allergy history, and signals from wearable devices or health-tracking apps. The AI expert emphasized that building a personalized nutrition model demands a precise understanding of nutritional science, human physiology, and high-quality training data. However, given that chatbots might make mistakes such as recommending diets containing allergens [31] or harmful diet tips that promote eating disorders [32], they should be regarded as supplementary tools to human nutritionists rather than as their replacements.

### Tracking and Other Alternatives

Responses from the physiotherapist and the AI expert to this topic largely echoed those provided during the “physical therapy” round. The athlete noted that the automated tracking, recording, and reminding function helps reduce stress, echoing the psychologist’s comments. In line with remarks made by other researchers [33], the simulation highlighted several advantages of chatbots over traditional methods in sports medicine. These included reducing the need for manual reporting, offering convenient cloud-based access to records, real-time data collection, instantaneous analysis, and providing immediate advice. Despite these benefits, the simulation lacked a discussion on how chatbots could potentially enhance treatment outcomes over alternative tools such as increasing patient satisfaction or reducing the recovery duration. In addition, the questions were designed to invoke engagement from all panelists. However, the nutritionist unexpectedly did not respond (Table 1).

### Ethics

Distinct from other audience-initiated topics, questions regarding ethics prompted responses from all panelists (Table 1). Some comments reiterated points from previous discussions, particularly regarding patient education. The conversation emphasized the need for stringent adherence to medical privacy regulations such as the Health Insurance Portability and Accountability Act in the United States or the General Data Protection Regulation in Europe [34]. This discussion highlighted the necessity of robust protocols for data encryption and storage to ensure security, as well as the need for transparency on data collection, processing, and accessibility. However, the panel did not delve into the merits and drawbacks of open-source, locally deployed chatbots (especially those furnished with domain-specific knowledge) versus commercial and online chatbots about privacy and security [35].

Regarding bias and fairness, it was stressed that chatbot training should use diverse and representative datasets. As users, athletes should retain complete discretion on whether to use chatbots, alternative methods, or a combination of both. The psychologist highlighted the need to implement chatbots in a manner that avoids triggering anxiety or other negative emotions. All the comments align with the 5 ethical principles proposed by AI4People: beneficence, nonmaleficence, justice, autonomy, and explicability [36].

### Closing Question

The moderator was prompted to steer the panel discussion toward its end with a final question. As anticipated, the questions were all forward-thinking (Multimedia Appendix 1). Panelists offered predictions drawing from their respective fields of expertise. Foreseeing rapid advancements in AI and complementary technologies, the panel envisaged a future of precision sports rehabilitation in the chatbot era. In this vision, the rehabilitation program would be tailored to individual needs, bolstered by health care providers, and empowered by chatbots. According to responses from the simulated athlete, this form of personalized support would make rehabilitation feel like a natural part of the recovery process, and the athlete would take charge of the rehabilitation journey.

## Discussion

### Principal Findings

We evaluated ChatGPT’s competency in addressing interdisciplinary inquiry using sports rehabilitation as an example. Using a novel model named PanelGPT, we prompted ChatGPT to explore the pros and cons of chatbot use in sports rehabilitation. ChatGPT answered questions via a simulated panel discussion where it role-played multiple experts, including a physiotherapist, psychologist, nutritionist, AI expert, and athlete. Our analysis of its responses highlighted benefits such as 24/7 support, personalized advice, and automated tracking, as well as challenges such as limited interaction modes, inaccuracies in emotion-related advice, and data privacy concerns. We repeated the experiments with the most recent version, GPT-4o (May 2024), and obtained generally similar results. Thus, our findings highlight the potential of using ChatGPT through PanelGPT to enhance appreciation of any interdisciplinary topic.

The interdisciplinary approach through PanelGPT brings several benefits with significant implications in medical education. First, the responses come from a panelist of experts with complementary expertise, providing different perspectives that are automatically categorized and offering a comprehensive view of the topic in question. For instance, including an athlete on the panel yielded a unique user perspective that could be overlooked in simple prompts. For example, a simple prompt of the questions on “psychological support” to ChatGPT yielded responses rooted in knowledge based on a psychologist (Multimedia Appendix 6). Thus, PanelGPT can offer students a holistic view of a complex interdisciplinary topic and integrates insights that might be missed from traditional educational settings.

Second, as LLMs become increasingly adapted in education, it is important to educate students on alternative, innovative ways of using chatbots. Compared to conventional communication with a chatbot, PanelGPT is novel in that it focuses the chatbot’s attention on the question and provides critical contexts for responding to the questions. For instance, when the “physical therapy” questions were simply prompted to ChatGPT, the responses quickly drifted toward other topics such as education and mental health (see Multimedia Appendix 7). With PanelGPT, the response involved a discussion between a physiotherapist and an AI expert, and the topic remained in the context of sports rehabilitation.

Third, the multirole-playing feature of ChatGPT through PanelGPT makes learning more interactive and engaging by encouraging active participation from learners. It also helps learners develop critical thinking skills such as synthesizing information from multiple simulated experts from different backgrounds and evaluating their credibility. This is particularly important when addressing the pros and cons of implementing new technologies in health care settings on topics that are interdisciplinary by nature.

Finally, having a panel of experts enables students to form a balanced view on a specific topic. For example, in addressing the “physical therapy” questions, the physiologist’s response highlighted the current limitations of chatbots in text or voice communication, while the AI expert expanded the discussion to the integration of real-time video analysis (Multimedia Appendices 3-5). This balanced view is crucial in medical education, as it allows students to understand both the potential and the limitations of any emerging technology (such as chatbots) that are poised for health care applications.

### Limitations

The breadth and depth of a panelist’s response depend on the training dataset in the field. In several discussions, such as the “patient education” and “tracking and other alternatives” topics, where we expected feedback from all panelists, there was a noticeable lack of direct responses from the nutritionist. It could be that the dataset used to train ChatGPT for the nutritionist was underrepresented in the rehabilitation field. Indeed, a combined search for “rehabilitation” (or “rehab”) and “nutritionist” (or “nutrition”) on PubMed yielded 6-8 times fewer hits compared to searches involving the terms “physiotherapist” (or “physiotherapy”) or “psychologist” (or “psychology”) (as of July 7, 2023). To address this limitation, the human operator could send reminders to the nutritionist to elicit a response. In contrast to the nutritionist, the AI expert responded to questions on all topics. This is expected because of the inherent need for AI expertise in creating such chatbot systems.

The data used to train ChatGPT at the time of our experiments only extended up to September 2021. As such, ChatGPT could not provide comments that would reference more recent developments in chatbots such as ChatGPT itself or BARD (now known as Gemini). The feature to activate Bing within ChatGPT does allow for real-time information browsing from the internet. However, in practice, this disrupted the panel discussion’s flow, resulting in a shift back to the regular ChatGPT conversation format and a subsequent loss of the expert identities after several exchanges (as shown in Multimedia Appendices 8-10).

We observed instances where the response to a question from the same expert was vague in one simulation but detailed in another. This observation suggests that conducting multiple simulations could enhance the efficacy of PanelGPT in providing a well-rounded understanding of the knowledge landscape surrounding an interdisciplinary topic. This practice enables self-consistency checking, which has been shown to improve the reasoning performance of language models [17]. Additionally, summarizing diverse responses from multiple simulations facilitates the identification of contrasting viewpoints and emergent trends in the panel discussion.

Hallucination, the generation of unsupported or false information, is a prevalent issue with LLM-based chatbots. The multiperspective approach of PanelGPT allows the chatbot to draw on the strengths and mitigate the weaknesses of each panelist when responding to specific questions. The current model is constrained by the same chatbot simulating all the panelists in a given chat session. With advances in chatbot development, this model could be extended by integrating responses from other LLM chatbots, especially those possessing domain-specific knowledge. In fact, cross-referencing responses from different experts on the panel powered by distinct models helps mitigate hallucination [37]. Nonetheless, it remains crucial to cross-verify the conclusions drawn from the simulation with literature findings or opinions from human experts to ensure the accuracy of the information.

Throughout the simulation, we noted instances where comments from one expert were acknowledged by another. Intriguingly, contradictory comments between experts were not observed. The richness and depth of the discussion can be further enhanced by using additional prompting strategies. For instance, after each response round, panelists could be prompted to critically evaluate each other’s comments to foster consensus or highlight disagreements. Panelists may also be prompted to pose questions to one another, such as seeking clarifications or requesting further details on a given response. Moreover, panelists could prompt the audience to clarify their questions if necessary. These additional prompting tactics make the panel discussion more engaging and mirror a real-life scenario, increasing the likelihood of obtaining a thorough appreciation of the topic.

### Conclusions

We presented PanelGPT, an innovative method that capitalizes on the multirole-playing feature of ChatGPT through simulated panel discussions, and applied it to evaluate ChatGPT’s competency in addressing interdisciplinary inquiry. In our case study, ChatGPT adequately addressed the opportunities and challenges on chatbot uses in sports rehabilitation. As a generalizable model, we envision PanelGPT as a supplementary tool in the classroom, aiding students in understanding complex interdisciplinary topics in medical education, such as nursing care, sports rehabilitation, stroke rehabilitation, and the management of recurrent pneumonia in long-term care facilities.

## Appendix

Multimedia Appendix 1 Questions designed for the simulated panel discussion on “chatbots in sports rehabilitation.”.

Multimedia Appendix 2 Prompts used to steer the simulated panel discussion.

Multimedia Appendix 3 Prompts and scripts for the 1st round of simulation.

Multimedia Appendix 4 Prompts and scripts for the 2nd round of simulation.

Multimedia Appendix 5 Prompts and scripts for the 3rd round of simulation.

Multimedia Appendix 6 Scripts for a direct prompt on "psychological support.".

Multimedia Appendix 7 Scripts for a direct prompt on "physical therapy.".

Multimedia Appendix 8 Prompts and scripts for the 1st round of simulation with Bing activated.

Multimedia Appendix 9 Prompts and scripts for the 2nd round of simulation with Bing activated.

Multimedia Appendix 10 Prompts and scripts for the 3rd round of simulation with Bing activated.

## Abbreviations

AI: artificial intelligence
LLM: large language model
