# Supplementary material for: Assessing ChatGPT’s Competency in Addressing Interdisciplinary Inquiries on Chatbot Uses in Sports Rehabilitation: Simulation Study
Source: JMIR Med Educ. 2024 Aug 7;10:e51157. doi: 10.2196/51157 (PMC11339563; doi:10.2196/51157)
Supplement: Multimedia Appendix 1 [file mededu_v10i1e51157_app1.docx]

**Multimedia Appendix 1:** Questions designed for the simulated panel discussion on “chatbots in sports rehabilitation”.

| **Category** | **Questions** | **Source** |
| --- | --- | --- |
| Opening Question | What role do you see chatbots playing in sports rehabilitation currently? And how do you see this role evolving in the future? | Directly from ChatGPT-4 |
| Patient Education | What education should we offer to guide our athlete the use of a chatbot for rehab? | From authors, but inspired by pilot simulations with ChatGPT-4 |
| Physical Therapy | How can chatbots help in an athlete's recovery? Can they really understand things like how the athlete moves, or how they distribute their weight, and then give specific advice about exercises that are just right for them? | From authors, inspired by Cheng et al [15] ; polished by ChatGPT-4 into oral English |
| Psychological Support | How are chatbots improving the psychological health of injured athletes? Can they really pick up on the emotional tone in our words and adjust their responses to suit our mood? | From authors, inspired by Oh et al [16]; polished by ChatGPT-4 into oral English |
| Nutrition | How can chatbots improve in nutrition management for athletes during recovery? | From authors, but inspired by pilot simulations with ChatGPT-4 |
| Tracking and Other Alternatives | How do chatbots keep track of an athlete's recovery? And do they have any special advantages over the old ways we used to do this? | From authors, inspired by Dwyer et al [14]; polished by ChatGPT-4 into oral English |
| Ethics | When we bring chatbots into athlete recovery, how do we make sure we respect patient choice, keep data private and safe? How do we handle worries about things like bias, fairness, and not undervaluing the human touch in our clinics? | From authors, *ad hoc*; polished by ChatGPT-4 into oral English |
| Closing Question | 1: Given the rapidly evolving capabilities of AI and chatbots, where do you see the future of chatbots in sports rehabilitation, say in the next 5 to 10 years? 2: As we look forward to the future, what is one exciting opportunity or development that you hope to see in the use of chatbots in sports rehabilitation? 3: What do you each believe is the most exciting opportunity or development for the use of chatbots in sports rehabilitation in the near future? | Directly from ChatGPT-4 |
