# Supplementary material for: Assessing ChatGPT’s Competency in Addressing Interdisciplinary Inquiries on Chatbot Uses in Sports Rehabilitation: Simulation Study
Source: JMIR Med Educ. 2024 Aug 7;10:e51157. doi: 10.2196/51157 (PMC11339563; doi:10.2196/51157)
Supplement: Multimedia Appendix 2 [file mededu_v10i1e51157_app2.docx]

**Multimedia Appendix 2:** Prompts used to steer the simulated panel discussion.

| Introductory prompts*   - You will assume various roles in a thought-provoking panel on "Chatbots in Sports Rehabilitation: Opportunities and Challenges." The simulation will involve a skilled moderator, Henry, who will guide the discussion, along with esteemed professionals in their respective fields: Dr. Mandy, a sports physiotherapist; Dr. Perry, a sports psychologist; Nikki a sports nutritionist; Allan, a professional athlete who had successfully navigated the rehabilitation process after a severe injury; and Nelson, an expert in Natural Language Processing for clinical application. I will role-play the audience and send you questions or reminders. While multiple experts can offer their insights, no question should be left unanswered. When replying to a question, please use spoken English. Henry, please begin the panel discussion by introducing the panel, followed by asking the panel about the role of chatbots in sports rehabilitation and how they see this role evolving. |
| --- |
| Inquiry prompts   - The same as the “Questions” column in **Table 1**. |
| Other prompts   - Henry, would you summarize and open the floor for questions? - Henry, would you summarize the response and move to solicit the next question? - Experts on the panel, please provide comments on the question. - Henry, would you summarize the response? After that, because the time is up, please think of one last question to ask the panel to conclude the discussion. - Henry, please conclude the panel discussion and ask the audience for another round of applause to thank the panel. |

*To integrate BING search into the simulation, activate BING in the ChatGPT settings. Subsequently, add the sentence, “A BING search may be performed in order to answer each of the questions with the most up to date information” after the statement, “While multiple experts can offer their insights, no question should be left unanswered.” We observed that use of the BING feature frequently disrupted the panel discussion's flow, causing it to revert to a regular ChatGPT conversation (see discussion).
